# Supplementary material for: The effects of genital myiasis on the diversity of the vaginal microbiota in female Bactrian camels
Source: BMC Vet Res. 2022 Mar 5;18:87. doi: 10.1186/s12917-022-03189-5 (PMC8897907; doi:10.1186/s12917-022-03189-5)
Supplement: Supplementary file 5 — Additional file 5. [file 12917_2022_3189_MOESM5_ESM.zip › MPL201709200_16s_yy/Treat1/css/js_css/jsPDF-master.zip!/jsPDF-master/docs/plugins_from_html.js.html]

plugins/from\_html.js - Documentation


## Home

### Classes

- jsPDF

### Global

- addFont
- addHTML
- addMetadata
- addPage
- autoPrint
- CapJoinStyles
- circle
- ellipse
- getFontList
- lines
- lstext
- output
- rect
- roundedRect
- save
- setDisplayMode
- setDrawColor
- setFillColor
- setFont
- setFontSize
- setFontStyle
- setLineCap
- setLineJoin
- setLineWidth
- setPage
- setProperties
- setTextColor
- text
- triangle

# plugins/from\_html.js

```
/** @preserve
 * jsPDF fromHTML plugin. BETA stage. API subject to change. Needs browser
 * Copyright (c) 2012 Willow Systems Corporation, willow-systems.com
 *               2014 Juan Pablo Gaviria, https://github.com/juanpgaviria
 *               2014 Diego Casorran, https://github.com/diegocr
 *               2014 Daniel Husar, https://github.com/danielhusar
 *               2014 Wolfgang Gassler, https://github.com/woolfg
 *               2014 Steven Spungin, https://github.com/flamenco
 *
 * Permission is hereby granted, free of charge, to any person obtaining
 * a copy of this software and associated documentation files (the
 * "Software"), to deal in the Software without restriction, including
 * without limitation the rights to use, copy, modify, merge, publish,
 * distribute, sublicense, and/or sell copies of the Software, and to
 * permit persons to whom the Software is furnished to do so, subject to
 * the following conditions:
 *
 * The above copyright notice and this permission notice shall be
 * included in all copies or substantial portions of the Software.
 *
 * THE SOFTWARE IS PROVIDED "AS IS", WITHOUT WARRANTY OF ANY KIND,
 * EXPRESS OR IMPLIED, INCLUDING BUT NOT LIMITED TO THE WARRANTIES OF
 * MERCHANTABILITY, FITNESS FOR A PARTICULAR PURPOSE AND
 * NONINFRINGEMENT. IN NO EVENT SHALL THE AUTHORS OR COPYRIGHT HOLDERS BE
 * LIABLE FOR ANY CLAIM, DAMAGES OR OTHER LIABILITY, WHETHER IN AN ACTION
 * OF CONTRACT, TORT OR OTHERWISE, ARISING FROM, OUT OF OR IN CONNECTION
 * WITH THE SOFTWARE OR THE USE OR OTHER DEALINGS IN THE SOFTWARE.
 * ====================================================================
 */

(function (jsPDFAPI) {
	var clone,
	DrillForContent,
	FontNameDB,
	FontStyleMap,
	TextAlignMap,
	FontWeightMap,
	FloatMap,
	ClearMap,
	GetCSS,
	PurgeWhiteSpace,
	Renderer,
	ResolveFont,
	ResolveUnitedNumber,
	UnitedNumberMap,
	elementHandledElsewhere,
	images,
	loadImgs,
	checkForFooter,
	process,
	tableToJson;
	clone = (function () {
		return function (obj) {
			Clone.prototype = obj;
			return new Clone()
		};
		function Clone() {}
	})();
	PurgeWhiteSpace = function (array) {
		var fragment,
		i,
		l,
		lTrimmed,
		r,
		rTrimmed,
		trailingSpace;
		i = 0;
		l = array.length;
		fragment = void 0;
		lTrimmed = false;
		rTrimmed = false;
		while (!lTrimmed && i !== l) {
			fragment = array[i] = array[i].trimLeft();
			if (fragment) {
				lTrimmed = true;
			}
			i++;
		}
		i = l - 1;
		while (l && !rTrimmed && i !== -1) {
			fragment = array[i] = array[i].trimRight();
			if (fragment) {
				rTrimmed = true;
			}
			i--;
		}
		r = /\s+$/g;
		trailingSpace = true;
		i = 0;
		while (i !== l) {
			// Leave the line breaks intact
			if (array[i] != "\u2028") {
				fragment = array[i].replace(/\s+/g, " ");
				if (trailingSpace) {
					fragment = fragment.trimLeft();
				}
				if (fragment) {
					trailingSpace = r.test(fragment);
				}
				array[i] = fragment;
			}
			i++;
		}
		return array;
	};
	Renderer = function (pdf, x, y, settings) {
		this.pdf = pdf;
		this.x = x;
		this.y = y;
		this.settings = settings;
		//list of functions which are called after each element-rendering process
		this.watchFunctions = [];
		this.init();
		return this;
	};
	ResolveFont = function (css_font_family_string) {
		var name,
		part,
		parts;
		name = void 0;
		parts = css_font_family_string.split(",");
		part = parts.shift();
		while (!name && part) {
			name = FontNameDB[part.trim().toLowerCase()];
			part = parts.shift();
		}
		return name;
	};
	ResolveUnitedNumber = function (css_line_height_string) {

		//IE8 issues
		css_line_height_string = css_line_height_string === "auto" ? "0px" : css_line_height_string;
		if (css_line_height_string.indexOf("em") > -1 && !isNaN(Number(css_line_height_string.replace("em", "")))) {
			css_line_height_string = Number(css_line_height_string.replace("em", "")) * 18.719 + "px";
		}
		if (css_line_height_string.indexOf("pt") > -1 && !isNaN(Number(css_line_height_string.replace("pt", "")))) {
			css_line_height_string = Number(css_line_height_string.replace("pt", "")) * 1.333 + "px";
		}

		var normal,
		undef,
		value;
		undef = void 0;
		normal = 16.00;
		value = UnitedNumberMap[css_line_height_string];
		if (value) {
			return value;
		}
		value = {
			"xx-small"  :  9,
			"x-small"   : 11,
			small       : 13,
			medium      : 16,
			large       : 19,
			"x-large"   : 23,
			"xx-large"  : 28,
			auto        :  0
		}[{ css_line_height_string : css_line_height_string }];

		if (value !== undef) {
			return UnitedNumberMap[css_line_height_string] = value / normal;
		}
		if (value = parseFloat(css_line_height_string)) {
			return UnitedNumberMap[css_line_height_string] = value / normal;
		}
		value = css_line_height_string.match(/([\d\.]+)(px)/);
		if (value.length === 3) {
			return UnitedNumberMap[css_line_height_string] = parseFloat(value[1]) / normal;
		}
		return UnitedNumberMap[css_line_height_string] = 1;
	};
	GetCSS = function (element) {
		var css,tmp,computedCSSElement;
		computedCSSElement = (function (el) {
			var compCSS;
			compCSS = (function (el) {
				if (document.defaultView && document.defaultView.getComputedStyle) {
					return document.defaultView.getComputedStyle(el, null);
				} else if (el.currentStyle) {
					return el.currentStyle;
				} else {
					return el.style;
				}
			})(el);
			return function (prop) {
				prop = prop.replace(/-\D/g, function (match) {
					return match.charAt(1).toUpperCase();
				});
				return compCSS[prop];
			};
		})(element);
		css = {};
		tmp = void 0;
		css["font-family"] = ResolveFont(computedCSSElement("font-family")) || "times";
		css["font-style"] = FontStyleMap[computedCSSElement("font-style")] || "normal";
		css["text-align"] = TextAlignMap[computedCSSElement("text-align")] || "left";
		tmp = FontWeightMap[computedCSSElement("font-weight")] || "normal";
		if (tmp === "bold") {
			if (css["font-style"] === "normal") {
				css["font-style"] = tmp;
			} else {
				css["font-style"] = tmp + css["font-style"];
			}
		}
		css["font-size"] = ResolveUnitedNumber(computedCSSElement("font-size")) || 1;
		css["line-height"] = ResolveUnitedNumber(computedCSSElement("line-height")) || 1;
		css["display"] = (computedCSSElement("display") === "inline" ? "inline" : "block");

		tmp = (css["display"] === "block");
		css["margin-top"]     = tmp && ResolveUnitedNumber(computedCSSElement("margin-top"))     || 0;
		css["margin-bottom"]  = tmp && ResolveUnitedNumber(computedCSSElement("margin-bottom"))  || 0;
		css["padding-top"]    = tmp && ResolveUnitedNumber(computedCSSElement("padding-top"))    || 0;
		css["padding-bottom"] = tmp && ResolveUnitedNumber(computedCSSElement("padding-bottom")) || 0;
		css["margin-left"]    = tmp && ResolveUnitedNumber(computedCSSElement("margin-left"))    || 0;
		css["margin-right"]   = tmp && ResolveUnitedNumber(computedCSSElement("margin-right"))   || 0;
		css["padding-left"]   = tmp && ResolveUnitedNumber(computedCSSElement("padding-left"))   || 0;
		css["padding-right"]  = tmp && ResolveUnitedNumber(computedCSSElement("padding-right"))  || 0;

		css["page-break-before"] = computedCSSElement("page-break-before") || "auto";

		//float and clearing of floats
		css["float"] = FloatMap[computedCSSElement("cssFloat")] || "none";
		css["clear"] = ClearMap[computedCSSElement("clear")] || "none";

		css["color"]  = computedCSSElement("color");

		return css;
	};
	elementHandledElsewhere = function (element, renderer, elementHandlers) {
		var handlers,
		i,
		isHandledElsewhere,
		l,
		t;
		isHandledElsewhere = false;
		i = void 0;
		l = void 0;
		t = void 0;
		handlers = elementHandlers["#" + element.id];
		if (handlers) {
			if (typeof handlers === "function") {
				isHandledElsewhere = handlers(element, renderer);
			} else {
				i = 0;
				l = handlers.length;
				while (!isHandledElsewhere && i !== l) {
					isHandledElsewhere = handlers[i](element, renderer);
					i++;
				}
			}
		}
		handlers = elementHandlers[element.nodeName];
		if (!isHandledElsewhere && handlers) {
			if (typeof handlers === "function") {
				isHandledElsewhere = handlers(element, renderer);
			} else {
				i = 0;
				l = handlers.length;
				while (!isHandledElsewhere && i !== l) {
					isHandledElsewhere = handlers[i](element, renderer);
					i++;
				}
			}
		}
		return isHandledElsewhere;
	};
	tableToJson = function (table, renderer) {
		var data,
		headers,
		i,
		j,
		rowData,
		tableRow,
		table_obj,
		table_with,
		cell,
		l;
		data = [];
		headers = [];
		i = 0;
		l = table.rows[0].cells.length;
		table_with = table.clientWidth;
		while (i < l) {
			cell = table.rows[0].cells[i];
			headers[i] = {
				name : cell.textContent.toLowerCase().replace(/\s+/g, ''),
				prompt : cell.textContent.replace(/\r?\n/g, ''),
				width : (cell.clientWidth / table_with) * renderer.pdf.internal.pageSize.width
			};
			i++;
		}
		i = 1;
		while (i < table.rows.length) {
			tableRow = table.rows[i];
			rowData = {};
			j = 0;
			while (j < tableRow.cells.length) {
				rowData[headers[j].name] = tableRow.cells[j].textContent.replace(/\r?\n/g, '');
				j++;
			}
			data.push(rowData);
			i++;
		}
		return table_obj = {
			rows : data,
			headers : headers
		};
	};
	var SkipNode = {
		SCRIPT   : 1,
		STYLE    : 1,
		NOSCRIPT : 1,
		OBJECT   : 1,
		EMBED    : 1,
		SELECT   : 1
	};
	var listCount = 1;
	DrillForContent = function (element, renderer, elementHandlers) {
		var cn,
		cns,
		fragmentCSS,
		i,
		isBlock,
		l,
		px2pt,
		table2json,
		cb;
		cns = element.childNodes;
		cn = void 0;
		fragmentCSS = GetCSS(element);
		isBlock = fragmentCSS.display === "block";
		if (isBlock) {
			renderer.setBlockBoundary();
			renderer.setBlockStyle(fragmentCSS);
		}
		px2pt = 0.264583 * 72 / 25.4;
		i = 0;
		l = cns.length;
		while (i < l) {
			cn = cns[i];
			if (typeof cn === "object") {

				//execute all watcher functions to e.g. reset floating
				renderer.executeWatchFunctions(cn);

				/*** HEADER rendering **/
				if (cn.nodeType === 1 && cn.nodeName === 'HEADER') {
					var header = cn;
					//store old top margin
					var oldMarginTop = renderer.pdf.margins_doc.top;
					//subscribe for new page event and render header first on every page
					renderer.pdf.internal.events.subscribe('addPage', function (pageInfo) {
						//set current y position to old margin
						renderer.y = oldMarginTop;
						//render all child nodes of the header element
						DrillForContent(header, renderer, elementHandlers);
						//set margin to old margin + rendered header + 10 space to prevent overlapping
						//important for other plugins (e.g. table) to start rendering at correct position after header
						renderer.pdf.margins_doc.top = renderer.y + 10;
						renderer.y += 10;
					}, false);
				}

				if (cn.nodeType === 8 && cn.nodeName === "#comment") {
					if (~cn.textContent.indexOf("ADD_PAGE")) {
						renderer.pdf.addPage();
						renderer.y = renderer.pdf.margins_doc.top;
					}

				} else if (cn.nodeType === 1 && !SkipNode[cn.nodeName]) {
					/*** IMAGE RENDERING ***/
					var cached_image;
					if (cn.nodeName === "IMG") {
						var url = cn.getAttribute("src");
						cached_image = images[renderer.pdf.sHashCode(url) || url];
					}
					if (cached_image) {
						if ((renderer.pdf.internal.pageSize.height - renderer.pdf.margins_doc.bottom < renderer.y + cn.height) && (renderer.y > renderer.pdf.margins_doc.top)) {
							renderer.pdf.addPage();
							renderer.y = renderer.pdf.margins_doc.top;
							//check if we have to set back some values due to e.g. header rendering for new page
							renderer.executeWatchFunctions(cn);
						}

						var imagesCSS = GetCSS(cn);
						var imageX = renderer.x;
						var fontToUnitRatio = 12 / renderer.pdf.internal.scaleFactor;

						//define additional paddings, margins which have to be taken into account for margin calculations
						var additionalSpaceLeft = (imagesCSS["margin-left"] + imagesCSS["padding-left"])*fontToUnitRatio;
						var additionalSpaceRight = (imagesCSS["margin-right"] + imagesCSS["padding-right"])*fontToUnitRatio;
						var additionalSpaceTop = (imagesCSS["margin-top"] + imagesCSS["padding-top"])*fontToUnitRatio;
						var additionalSpaceBottom = (imagesCSS["margin-bottom"] + imagesCSS["padding-bottom"])*fontToUnitRatio;

						//if float is set to right, move the image to the right border
						//add space if margin is set
						if (imagesCSS['float'] !== undefined && imagesCSS['float'] === 'right') {
							imageX += renderer.settings.width - cn.width - additionalSpaceRight;
						} else {
							imageX +=  additionalSpaceLeft;
						}

						renderer.pdf.addImage(cached_image, imageX, renderer.y + additionalSpaceTop, cn.width, cn.height);
						cached_image = undefined;
						//if the float prop is specified we have to float the text around the image
						if (imagesCSS['float'] === 'right' || imagesCSS['float'] === 'left') {
							//add functiont to set back coordinates after image rendering
							renderer.watchFunctions.push((function(diffX , thresholdY, diffWidth, el) {
								//undo drawing box adaptions which were set by floating
								if (renderer.y >= thresholdY) {
									renderer.x += diffX;
									renderer.settings.width += diffWidth;
									return true;
								} else if(el && el.nodeType === 1 && !SkipNode[el.nodeName] && renderer.x+el.width > (renderer.pdf.margins_doc.left + renderer.pdf.margins_doc.width)) {
									renderer.x += diffX;
									renderer.y = thresholdY;
									renderer.settings.width += diffWidth;
									return true;
								} else {
									return false;
								}
							}).bind(this, (imagesCSS['float'] === 'left') ? -cn.width-additionalSpaceLeft-additionalSpaceRight : 0, renderer.y+cn.height+additionalSpaceTop+additionalSpaceBottom, cn.width));
							//reset floating by clear:both divs
							//just set cursorY after the floating element
							renderer.watchFunctions.push((function(yPositionAfterFloating, pages, el) {
								if (renderer.y < yPositionAfterFloating && pages === renderer.pdf.internal.getNumberOfPages()) {
									if (el.nodeType === 1 && GetCSS(el).clear === 'both') {
										renderer.y = yPositionAfterFloating;
										return true;
									} else {
										return false;
									}
								} else {
									return true;
								}
							}).bind(this, renderer.y+cn.height, renderer.pdf.internal.getNumberOfPages()));

							//if floating is set we decrease the available width by the image width
							renderer.settings.width -= cn.width+additionalSpaceLeft+additionalSpaceRight;
							//if left just add the image width to the X coordinate
							if (imagesCSS['float'] === 'left') {
								renderer.x += cn.width+additionalSpaceLeft+additionalSpaceRight;
							}
						} else {
						//if no floating is set, move the rendering cursor after the image height
							renderer.y += cn.height + additionalSpaceTop + additionalSpaceBottom;
						}

					/*** TABLE RENDERING ***/
					} else if (cn.nodeName === "TABLE") {
						table2json = tableToJson(cn, renderer);
						renderer.y += 10;
						renderer.pdf.table(renderer.x, renderer.y, table2json.rows, table2json.headers, {
							autoSize : false,
							printHeaders: elementHandlers.printHeaders,
							margins: renderer.pdf.margins_doc,
							css: GetCSS(cn)
						});
						renderer.y = renderer.pdf.lastCellPos.y + renderer.pdf.lastCellPos.h + 20;
					} else if (cn.nodeName === "OL" || cn.nodeName === "UL") {
						listCount = 1;
						if (!elementHandledElsewhere(cn, renderer, elementHandlers)) {
							DrillForContent(cn, renderer, elementHandlers);
						}
						renderer.y += 10;
					} else if (cn.nodeName === "LI") {
						var temp = renderer.x;
						renderer.x += 20 / renderer.pdf.internal.scaleFactor;
						renderer.y += 3;
						if (!elementHandledElsewhere(cn, renderer, elementHandlers)) {
							DrillForContent(cn, renderer, elementHandlers);
						}
						renderer.x = temp;
					} else if (cn.nodeName === "BR") {
						renderer.y += fragmentCSS["font-size"] * renderer.pdf.internal.scaleFactor;
						renderer.addText("\u2028", clone(fragmentCSS));
					} else {
						if (!elementHandledElsewhere(cn, renderer, elementHandlers)) {
							DrillForContent(cn, renderer, elementHandlers);
						}
					}
				} else if (cn.nodeType === 3) {
					var value = cn.nodeValue;
					if (cn.nodeValue && cn.parentNode.nodeName === "LI") {
						if (cn.parentNode.parentNode.nodeName === "OL") {
							value = listCount++ + '. ' + value;
						} else {
							var fontSize = fragmentCSS["font-size"];
							var offsetX = (3 - fontSize * 0.75) * renderer.pdf.internal.scaleFactor;
							var offsetY = fontSize * 0.75 * renderer.pdf.internal.scaleFactor;
							var radius = fontSize * 1.74 / renderer.pdf.internal.scaleFactor;
							cb = function (x, y) {
								this.pdf.circle(x + offsetX, y + offsetY, radius, 'FD');
							};
						}
					}
					// Only add the text if the text node is in the body element
					// Add compatibility with IE11
					if(!!(cn.ownerDocument.body.compareDocumentPosition(cn) & 16)){
						renderer.addText(value, fragmentCSS);
					}
				} else if (typeof cn === "string") {
					renderer.addText(cn, fragmentCSS);
				}
			}
			i++;
		}
		elementHandlers.outY = renderer.y;

		if (isBlock) {
			return renderer.setBlockBoundary(cb);
		}
	};
	images = {};
	loadImgs = function (element, renderer, elementHandlers, cb) {
		var imgs = element.getElementsByTagName('img'),
		l = imgs.length, found_images,
		x = 0;
		function done() {
			renderer.pdf.internal.events.publish('imagesLoaded');
			cb(found_images);
		}
		function loadImage(url, width, height) {
			if (!url)
				return;
			var img = new Image();
			found_images = ++x;
			img.crossOrigin = '';
			img.onerror = img.onload = function () {
				if(img.complete) {
					//to support data urls in images, set width and height
					//as those values are not recognized automatically
					if (img.src.indexOf('data:image/') === 0) {
						img.width = width || img.width || 0;
						img.height = height || img.height || 0;
					}
					//if valid image add to known images array
					if (img.width + img.height) {
						var hash = renderer.pdf.sHashCode(url) || url;
						images[hash] = images[hash] || img;
					}
				}
				if(!--x) {
					done();
				}
			};
			img.src = url;
		}
		while (l--)
			loadImage(imgs[l].getAttribute("src"),imgs[l].width,imgs[l].height);
		return x || done();
	};
	checkForFooter = function (elem, renderer, elementHandlers) {
		//check if we can found a <footer> element
		var footer = elem.getElementsByTagName("footer");
		if (footer.length > 0) {

			footer = footer[0];

			//bad hack to get height of footer
			//creat dummy out and check new y after fake rendering
			var oldOut = renderer.pdf.internal.write;
			var oldY = renderer.y;
			renderer.pdf.internal.write = function () {};
			DrillForContent(footer, renderer, elementHandlers);
			var footerHeight = Math.ceil(renderer.y - oldY) + 5;
			renderer.y = oldY;
			renderer.pdf.internal.write = oldOut;

			//add 20% to prevent overlapping
			renderer.pdf.margins_doc.bottom += footerHeight;

			//Create function render header on every page
			var renderFooter = function (pageInfo) {
				var pageNumber = pageInfo !== undefined ? pageInfo.pageNumber : 1;
				//set current y position to old margin
				var oldPosition = renderer.y;
				//render all child nodes of the header element
				renderer.y = renderer.pdf.internal.pageSize.height - renderer.pdf.margins_doc.bottom;
				renderer.pdf.margins_doc.bottom -= footerHeight;

				//check if we have to add page numbers
				var spans = footer.getElementsByTagName('span');
				for (var i = 0; i < spans.length; ++i) {
					//if we find some span element with class pageCounter, set the page
					if ((" " + spans[i].className + " ").replace(/[\n\t]/g, " ").indexOf(" pageCounter ") > -1) {
						spans[i].innerHTML = pageNumber;
					}
					//if we find some span element with class totalPages, set a variable which is replaced after rendering of all pages
					if ((" " + spans[i].className + " ").replace(/[\n\t]/g, " ").indexOf(" totalPages ") > -1) {
						spans[i].innerHTML = '###jsPDFVarTotalPages###';
					}
				}

				//render footer content
				DrillForContent(footer, renderer, elementHandlers);
				//set bottom margin to previous height including the footer height
				renderer.pdf.margins_doc.bottom += footerHeight;
				//important for other plugins (e.g. table) to start rendering at correct position after header
				renderer.y = oldPosition;
			};

			//check if footer contains totalPages which should be replace at the disoposal of the document
			var spans = footer.getElementsByTagName('span');
			for (var i = 0; i < spans.length; ++i) {
				if ((" " + spans[i].className + " ").replace(/[\n\t]/g, " ").indexOf(" totalPages ") > -1) {
					renderer.pdf.internal.events.subscribe('htmlRenderingFinished', renderer.pdf.putTotalPages.bind(renderer.pdf, '###jsPDFVarTotalPages###'), true);
				}
			}

			//register event to render footer on every new page
			renderer.pdf.internal.events.subscribe('addPage', renderFooter, false);
			//render footer on first page
			renderFooter();

			//prevent footer rendering
			SkipNode['FOOTER'] = 1;
		}
	};
	process = function (pdf, element, x, y, settings, callback) {
		if (!element)
			return false;
		if (typeof element !== "string" && !element.parentNode)
			element = '' + element.innerHTML;
		if (typeof element === "string") {
			element = (function (element) {
				var $frame,
				$hiddendiv,
				framename,
				visuallyhidden;
				framename = "jsPDFhtmlText" + Date.now().toString() + (Math.random() * 1000).toFixed(0);
				visuallyhidden = "position: absolute !important;" + "clip: rect(1px 1px 1px 1px); /* IE6, IE7 */" + "clip: rect(1px, 1px, 1px, 1px);" + "padding:0 !important;" + "border:0 !important;" + "height: 1px !important;" + "width: 1px !important; " + "top:auto;" + "left:-100px;" + "overflow: hidden;";
				$hiddendiv = document.createElement('div');
				$hiddendiv.style.cssText = visuallyhidden;
				$hiddendiv.innerHTML = "<iframe style=\"height:1px;width:1px\" name=\"" + framename + "\" />";
				document.body.appendChild($hiddendiv);
				$frame = window.frames[framename];
				$frame.document.open();
				$frame.document.writeln(element);
				$frame.document.close();
				return $frame.document.body;
			})(element.replace(/<\/?script[^>]*?>/gi, ''));
		}
		var r = new Renderer(pdf, x, y, settings), out;

		// 1. load images
		// 2. prepare optional footer elements
		// 3. render content
		loadImgs.call(this, element, r, settings.elementHandlers, function (found_images) {
			checkForFooter( element, r, settings.elementHandlers);
			DrillForContent(element, r, settings.elementHandlers);
			//send event dispose for final taks (e.g. footer totalpage replacement)
			r.pdf.internal.events.publish('htmlRenderingFinished');
			out = r.dispose();
			if (typeof callback === 'function') callback(out);
			else if (found_images) console.error('jsPDF Warning: rendering issues? provide a callback to fromHTML!');
		});
		return out || {x: r.x, y:r.y};
	};
	Renderer.prototype.init = function () {
		this.paragraph = {
			text : [],
			style : []
		};
		return this.pdf.internal.write("q");
	};
	Renderer.prototype.dispose = function () {
		this.pdf.internal.write("Q");
		return {
			x : this.x,
			y : this.y,
			ready:true
		};
	};

	//Checks if we have to execute some watcher functions
	//e.g. to end text floating around an image
	Renderer.prototype.executeWatchFunctions = function(el) {
		var ret = false;
		var narray = [];
		if (this.watchFunctions.length > 0) {
			for(var i=0; i< this.watchFunctions.length; ++i) {
				if (this.watchFunctions[i](el) === true) {
					ret = true;
				} else {
					narray.push(this.watchFunctions[i]);
				}
			}
			this.watchFunctions = narray;
		}
		return ret;
	};

	Renderer.prototype.splitFragmentsIntoLines = function (fragments, styles) {
		var currentLineLength,
		defaultFontSize,
		ff,
		fontMetrics,
		fontMetricsCache,
		fragment,
		fragmentChopped,
		fragmentLength,
		fragmentSpecificMetrics,
		fs,
		k,
		line,
		lines,
		maxLineLength,
		style;
		defaultFontSize = 12;
		k = this.pdf.internal.scaleFactor;
		fontMetricsCache = {};
		ff = void 0;
		fs = void 0;
		fontMetrics = void 0;
		fragment = void 0;
		style = void 0;
		fragmentSpecificMetrics = void 0;
		fragmentLength = void 0;
		fragmentChopped = void 0;
		line = [];
		lines = [line];
		currentLineLength = 0;
		maxLineLength = this.settings.width;
		while (fragments.length) {
			fragment = fragments.shift();
			style = styles.shift();
			if (fragment) {
				ff = style["font-family"];
				fs = style["font-style"];
				fontMetrics = fontMetricsCache[ff + fs];
				if (!fontMetrics) {
					fontMetrics = this.pdf.internal.getFont(ff, fs).metadata.Unicode;
					fontMetricsCache[ff + fs] = fontMetrics;
				}
				fragmentSpecificMetrics = {
					widths : fontMetrics.widths,
					kerning : fontMetrics.kerning,
					fontSize : style["font-size"] * defaultFontSize,
					textIndent : currentLineLength
				};
				fragmentLength = this.pdf.getStringUnitWidth(fragment, fragmentSpecificMetrics) * fragmentSpecificMetrics.fontSize / k;
				if (fragment == "\u2028") {
					line = [];
					lines.push(line);
				} else if (currentLineLength + fragmentLength > maxLineLength) {
					fragmentChopped = this.pdf.splitTextToSize(fragment, maxLineLength, fragmentSpecificMetrics);
					line.push([fragmentChopped.shift(), style]);
					while (fragmentChopped.length) {
						line = [[fragmentChopped.shift(), style]];
						lines.push(line);
					}
					currentLineLength = this.pdf.getStringUnitWidth(line[0][0], fragmentSpecificMetrics) * fragmentSpecificMetrics.fontSize / k;
				} else {
					line.push([fragment, style]);
					currentLineLength += fragmentLength;
				}
			}
		}

		//if text alignment was set, set margin/indent of each line
		if (style['text-align'] !== undefined && (style['text-align'] === 'center' || style['text-align'] === 'right' || style['text-align'] === 'justify')) {
			for (var i = 0; i < lines.length; ++i) {
				var length = this.pdf.getStringUnitWidth(lines[i][0][0], fragmentSpecificMetrics) * fragmentSpecificMetrics.fontSize / k;
				//if there is more than on line we have to clone the style object as all lines hold a reference on this object
				if (i > 0) {
					lines[i][0][1] = clone(lines[i][0][1]);
				}
				var space = (maxLineLength - length);

				if (style['text-align'] === 'right') {
					lines[i][0][1]['margin-left'] = space;
					//if alignment is not right, it has to be center so split the space to the left and the right
				} else if (style['text-align'] === 'center') {
					lines[i][0][1]['margin-left'] = space / 2;
					//if justify was set, calculate the word spacing and define in by using the css property
				} else if (style['text-align'] === 'justify') {
					var countSpaces = lines[i][0][0].split(' ').length - 1;
					lines[i][0][1]['word-spacing'] = space / countSpaces;
					//ignore the last line in justify mode
					if (i === (lines.length - 1)) {
						lines[i][0][1]['word-spacing'] = 0;
					}
				}
			}
		}

		return lines;
	};
	Renderer.prototype.RenderTextFragment = function (text, style) {
		var defaultFontSize,
		font,
		maxLineHeight;

		maxLineHeight = 0;
		defaultFontSize = 12;

		if (this.pdf.internal.pageSize.height - this.pdf.margins_doc.bottom < this.y + this.pdf.internal.getFontSize()) {
			this.pdf.internal.write("ET", "Q");
			this.pdf.addPage();
			this.y = this.pdf.margins_doc.top;
			this.pdf.internal.write("q", "BT 0 g", this.pdf.internal.getCoordinateString(this.x), this.pdf.internal.getVerticalCoordinateString(this.y), style.color, "Td");
			//move cursor by one line on new page
			maxLineHeight = Math.max(maxLineHeight, style["line-height"], style["font-size"]);
			this.pdf.internal.write(0, (-1 * defaultFontSize * maxLineHeight).toFixed(2), "Td");
		}

		font = this.pdf.internal.getFont(style["font-family"], style["font-style"]);

		// text color
		var pdfTextColor = this.getPdfColor(style["color"]);
		if (pdfTextColor !== this.lastTextColor)
		{
			this.pdf.internal.write(pdfTextColor);
			this.lastTextColor = pdfTextColor;
		}

		//set the word spacing for e.g. justify style
		if (style['word-spacing'] !== undefined && style['word-spacing'] > 0) {
			this.pdf.internal.write(style['word-spacing'].toFixed(2), "Tw");
		}

		this.pdf.internal.write("/" + font.id, (defaultFontSize * style["font-size"]).toFixed(2), "Tf", "(" + this.pdf.internal.pdfEscape(text) + ") Tj");


		//set the word spacing back to neutral => 0
		if (style['word-spacing'] !== undefined) {
			this.pdf.internal.write(0, "Tw");
		}
	};

	// Accepts #FFFFFF, rgb(int,int,int), or CSS Color Name
	Renderer.prototype.getPdfColor = function(style) {
		var textColor;
		var r,g,b;

		var rx = /rgb\s*\(\s*(\d+),\s*(\d+),\s*(\d+\s*)\)/;
		var m = rx.exec(style);
		if (m != null){
			r = parseInt(m[1]);
			g = parseInt(m[2]);
			b = parseInt(m[3]);
		}
		else{
			if (style.charAt(0) != '#') {
				style = CssColors.colorNameToHex(style);
				if (!style) {
					style = '#000000';
				}
			}
			r = style.substring(1, 3);
			r = parseInt(r, 16);
			g = style.substring(3, 5);
			g = parseInt(g, 16);
			b = style.substring(5, 7);
			b = parseInt(b, 16);
		}

		if ((typeof r === 'string') && /^#[0-9A-Fa-f]{6}$/.test(r)) {
			var hex = parseInt(r.substr(1), 16);
			r = (hex >> 16) & 255;
			g = (hex >> 8) & 255;
			b = (hex & 255);
		}

		var f3 = this.f3;
		if ((r === 0 && g === 0 && b === 0) || (typeof g === 'undefined')) {
			textColor = f3(r / 255) + ' g';
		} else {
			textColor = [f3(r / 255), f3(g / 255), f3(b / 255), 'rg'].join(' ');
		}
		return textColor;
	};

	Renderer.prototype.f3 = function(number) {
		return number.toFixed(3); // Ie, %.3f
	},


	Renderer.prototype.renderParagraph = function (cb) {
		var blockstyle,
		defaultFontSize,
		fontToUnitRatio,
		fragments,
		i,
		l,
		line,
		lines,
		maxLineHeight,
		out,
		paragraphspacing_after,
		paragraphspacing_before,
		priorblockstyle,
		styles,
		fontSize;
		fragments = PurgeWhiteSpace(this.paragraph.text);
		styles = this.paragraph.style;
		blockstyle = this.paragraph.blockstyle;
		priorblockstyle = this.paragraph.priorblockstyle || {};
		this.paragraph = {
			text : [],
			style : [],
			blockstyle : {},
			priorblockstyle : blockstyle
		};
		if (!fragments.join("").trim()) {
			return;
		}
		lines = this.splitFragmentsIntoLines(fragments, styles);
		line = void 0;
		maxLineHeight = void 0;
		defaultFontSize = 12;
		fontToUnitRatio = defaultFontSize / this.pdf.internal.scaleFactor;
		this.priorMarginBottom =  this.priorMarginBottom || 0;
		paragraphspacing_before = (Math.max((blockstyle["margin-top"] || 0) - this.priorMarginBottom, 0) + (blockstyle["padding-top"] || 0)) * fontToUnitRatio;
		paragraphspacing_after = ((blockstyle["margin-bottom"] || 0) + (blockstyle["padding-bottom"] || 0)) * fontToUnitRatio;
		this.priorMarginBottom =  blockstyle["margin-bottom"] || 0;

		if (blockstyle['page-break-before'] === 'always'){
			this.pdf.addPage();
			this.y = 0;
			paragraphspacing_before = ((blockstyle["margin-top"] || 0) + (blockstyle["padding-top"] || 0)) * fontToUnitRatio;
		}

		out = this.pdf.internal.write;
		i = void 0;
		l = void 0;
		this.y += paragraphspacing_before;
		out("q", "BT 0 g", this.pdf.internal.getCoordinateString(this.x), this.pdf.internal.getVerticalCoordinateString(this.y), "Td");

		//stores the current indent of cursor position
		var currentIndent = 0;

		while (lines.length) {
			line = lines.shift();
			maxLineHeight = 0;
			i = 0;
			l = line.length;
			while (i !== l) {
				if (line[i][0].trim()) {
					maxLineHeight = Math.max(maxLineHeight, line[i][1]["line-height"], line[i][1]["font-size"]);
					fontSize = line[i][1]["font-size"] * 7;
				}
				i++;
			}
			//if we have to move the cursor to adapt the indent
			var indentMove = 0;
			var wantedIndent = 0;
			//if a margin was added (by e.g. a text-alignment), move the cursor
			if (line[0][1]["margin-left"] !== undefined && line[0][1]["margin-left"] > 0) {
				wantedIndent = this.pdf.internal.getCoordinateString(line[0][1]["margin-left"]);
				indentMove = wantedIndent - currentIndent;
				currentIndent = wantedIndent;
			}
			var indentMore = (Math.max(blockstyle["margin-left"] || 0, 0)) * fontToUnitRatio;
			//move the cursor
			out(indentMove + indentMore, (-1 * defaultFontSize * maxLineHeight).toFixed(2), "Td");
			i = 0;
			l = line.length;
			while (i !== l) {
				if (line[i][0]) {
					this.RenderTextFragment(line[i][0], line[i][1]);
				}
				i++;
			}
			this.y += maxLineHeight * fontToUnitRatio;

			//if some watcher function was executed successful, so e.g. margin and widths were changed,
			//reset line drawing and calculate position and lines again
			//e.g. to stop text floating around an image
			if (this.executeWatchFunctions(line[0][1]) && lines.length > 0) {
				var localFragments = [];
				var localStyles = [];
				//create fragment array of
				lines.forEach(function(localLine) {
					var i = 0;
					var l = localLine.length;
					while (i !== l) {
						if (localLine[i][0]) {
							localFragments.push(localLine[i][0]+' ');
							localStyles.push(localLine[i][1]);
						}
						++i;
					}
				});
				//split lines again due to possible coordinate changes
				lines = this.splitFragmentsIntoLines(PurgeWhiteSpace(localFragments), localStyles);
				//reposition the current cursor
				out("ET", "Q");
				out("q", "BT 0 g", this.pdf.internal.getCoordinateString(this.x), this.pdf.internal.getVerticalCoordinateString(this.y), "Td");
			}

		}
		if (cb && typeof cb === "function") {
			cb.call(this, this.x - 9, this.y - fontSize / 2);
		}
		out("ET", "Q");
		return this.y += paragraphspacing_after;
	};
	Renderer.prototype.setBlockBoundary = function (cb) {
		return this.renderParagraph(cb);
	};
	Renderer.prototype.setBlockStyle = function (css) {
		return this.paragraph.blockstyle = css;
	};
	Renderer.prototype.addText = function (text, css) {
		this.paragraph.text.push(text);
		return this.paragraph.style.push(css);
	};
	FontNameDB = {
		helvetica         : "helvetica",
		"sans-serif"      : "helvetica",
		"times new roman" : "times",
		serif             : "times",
		times             : "times",
		monospace         : "courier",
		courier           : "courier"
	};
	FontWeightMap = {
		100 : "normal",
		200 : "normal",
		300 : "normal",
		400 : "normal",
		500 : "bold",
		600 : "bold",
		700 : "bold",
		800 : "bold",
		900 : "bold",
		normal  : "normal",
		bold    : "bold",
		bolder  : "bold",
		lighter : "normal"
	};
	FontStyleMap = {
		normal  : "normal",
		italic  : "italic",
		oblique : "italic"
	};
	TextAlignMap = {
		left    : "left",
		right   : "right",
		center  : "center",
		justify : "justify"
	};
	FloatMap = {
		none : 'none',
		right: 'right',
		left: 'left'
	};
	ClearMap = {
	  none : 'none',
	  both : 'both'
	};
	UnitedNumberMap = {
		normal : 1
	};
	/**
	 * Converts HTML-formatted text into formatted PDF text.
	 *
	 * Notes:
	 * 2012-07-18
	 * Plugin relies on having browser, DOM around. The HTML is pushed into dom and traversed.
	 * Plugin relies on jQuery for CSS extraction.
	 * Targeting HTML output from Markdown templating, which is a very simple
	 * markup - div, span, em, strong, p. No br-based paragraph separation supported explicitly (but still may work.)
	 * Images, tables are NOT supported.
	 *
	 * @public
	 * @function
	 * @param HTML {String or DOM Element} HTML-formatted text, or pointer to DOM element that is to be rendered into PDF.
	 * @param x {Number} starting X coordinate in jsPDF instance's declared units.
	 * @param y {Number} starting Y coordinate in jsPDF instance's declared units.
	 * @param settings {Object} Additional / optional variables controlling parsing, rendering.
	 * @returns {Object} jsPDF instance
	 */
	jsPDFAPI.fromHTML = function (HTML, x, y, settings, callback, margins) {
		"use strict";

		this.margins_doc = margins || {
			top : 0,
			bottom : 0
		};
		if (!settings)
			settings = {};
		if (!settings.elementHandlers)
			settings.elementHandlers = {};

		return process(this, HTML, isNaN(x) ? 4 : x, isNaN(y) ? 4 : y, settings, callback);
	};
})(jsPDF.API);
```

  

Documentation generated by JSDoc 3.4.2 on Sun Oct 09 2016 11:08:27 GMT+0100 (BST) using the docdash theme.
